# Trastuzumab, in combination with carboplatin and docetaxel, does not prolong the QT interval of patients with HER2-positive metastatic or locally advanced inoperable solid tumors: results from a phase Ib study

**DOI:** 10.1007/s00280-014-2603-9

**Published:** 2014-10-26

**Authors:** Na Xu, Charles H. Redfern, Michael Gordon, Stephen Eppler, Bert L. Lum, Caroline Trudeau

**Affiliations:** 1 Genentech, Inc., 1 DNA Way, South San Francisco, CA 94080 USA; 2 Sharp Healthcare, 3075 Health Center Drive, Suite 102, San Diego, CA 92123 USA; 3 Pinnacle Oncology Hematology, 9055 E. Del Camino, Suite 100, Scottsdale, AZ 85258 USA

**Keywords:** Carboplatin, Electrocardiogram, Human epidermal growth factor receptor 2, Trastuzumab, QT interval

## Abstract

**Purpose:**

This study evaluated the potential effect of trastuzumab on the electrocardiogram (ECG) QT interval and assessed the potential pharmacokinetic interaction between trastuzumab and carboplatin. Here, we report the QT and safety results.

**Methods:**

Patients with metastatic or inoperable HER2-positive solid tumors received docetaxel and carboplatin on Day 1 of each 3-week (q3w) cycle. Trastuzumab was administered intravenously, as an accelerated loading dose regimen, on Cycle 1, Day 2 and Cycle 1, Day 8, and then on Day 1 of each subsequent q3w cycle. ECG assessments were performed pre- and posttrastuzumab infusion in the first two cycles. Fridericia’s correction was applied to QT intervals (QTcF). Baseline-adjusted QTcF intervals (the change from baseline) and their 90 % confidence intervals (CIs) were calculated.

**Results:**

The study enrolled 59 patients. At all time points, the 90 % CI upper bound for the mean baseline-adjusted QTcF was <10 ms. At steady-state serum trastuzumab concentrations, the mean baseline-adjusted QTcF interval was −8.4 ms (90 % CI −11.1, −5.7). No patient exhibited an absolute QTcF interval of >480 ms. No relationship was observed between trastuzumab concentration and baseline-adjusted QTcF interval. At data cutoff, 84.5 % of patients had experienced grade ≥3 adverse events, the most common of which were hematologic and as expected. Left ventricular ejection fraction remained ≥45 % in all patients during the study.

**Conclusions:**

The results suggest that trastuzumab had no clinically relevant effect on QTcF interval. The safety profile of trastuzumab in combination with carboplatin and docetaxel was consistent with the known safety profile of this combination.

**Electronic supplementary material:**

The online version of this article (doi:10.1007/s00280-014-2603-9) contains supplementary material, which is available to authorized users.

## Introduction

Human epidermal growth factor receptor 2 (HER2) is amplified and/or overexpressed in 15–20 % of breast cancers [1] and in 10–24 % of gastric/gastroesophageal junction cancers [2, 3]. Patients with HER2-positive disease have a poorer prognosis compared with those with HER2-negative disease [4, 5]. The humanized monoclonal antibody trastuzumab (Herceptin^®^, F. Hoffmann-La Roche, Basel, Switzerland) binds to HER2, inhibiting ligand-independent HER2 signaling and promoting antibody-dependent cell-mediated cytotoxicity of HER2-positive cancer cells. Trastuzumab improves survival outcomes when added to chemotherapy for the treatment of patients with HER2-positive early breast cancer (EBC) [6], HER2-positive metastatic breast cancer (MBC) [7], and HER2-positive metastatic gastric or gastroesophageal junction cancer [8]. Therefore, trastuzumab is approved in the USA and EU for use in these settings [9, 10]. In addition to the originally approved intravenous formulation of trastuzumab, a subcutaneous formulation of trastuzumab was approved in the EU in 2013 for HER2-positive EBC and MBC [10].

Trastuzumab therapy has been linked to a low but clinically significant incidence of congestive heart failure, as well as decreased left ventricular ejection fraction (LVEF) [7, 11]. More broadly, a number of pharmaceutical agents have been reported to delay cardiac repolarization, a condition which favors the development of ventricular arrhythmia. For example, administration of docetaxel has been associated with a proarrhythmogenic effect [12]. Therefore, novel therapeutic agents should be evaluated for their potential to prolong the QT interval [as assessed by electrocardiogram (ECG)] [13]. Some HER2-targeted agents have been linked with QT prolongation. The HER2-targeted tyrosine kinase inhibitor lapatinib has been associated with prolongation of the corrected QT (QTc) interval from baseline to postbaseline time points in patients with advanced cancer [14]. In an observational study of patients with HER2-positive EBC treated with trastuzumab following an anthracycline-based regimen, a prolonged QT interval was observed compared with patients with HER2-negative EBC who were not treated with trastuzumab [15]. In contrast, a study of trastuzumab in patients with HER2-positive MBC revealed no abnormalities in atrial and ventricular depolarization and repolarization following trastuzumab infusion [16]. It should be noted that, due to their large molecular weight and high target specificity, monoclonal antibodies are not expected to bind to ion channels in the heart in the way that small molecules can; therefore, monoclonal antibodies are not expected to have an effect on the QT interval [17].

Here, we report results from a phase Ib study with two objectives: (1) to evaluate the potential effect of trastuzumab on the QTc interval, when combined with carboplatin and docetaxel; (2) to evaluate the potential for a pharmacokinetic drug–drug interaction between trastuzumab and carboplatin in the presence of docetaxel. The pharmacokinetic results are reported separately. This manuscript focuses on the QTc and safety results from the study.

## Methods

### Study design and objectives

This was a phase Ib, multicenter, single-arm, open-label study of trastuzumab in combination with carboplatin and docetaxel (Clinicaltrials.gov registration #NCT00927589). The primary ECG objective of the study was to investigate the effect of trastuzumab on the duration of the QT interval, corrected using Fridericia’s correction (QTcF), as measured by the change in mean QTcF interval from baseline to steady-state trastuzumab.

### Patient population

Patients with metastatic or locally advanced, inoperable, HER2-positive solid malignancies were eligible for the study. HER2-positivity for all tumor types was assessed with a testing kit that was FDA-approved for breast cancer or, if available, for the patient’s tumor type, including immunohistochemistry, fluorescence in situ hybridization, or chromogenic in situ hybridization assays, performed by a local or reference laboratory. Key exclusion criteria were as follows: an Eastern Cooperative Oncology Group performance status of >1, an LVEF of <50 % prior to Cycle 1, Day 1, a QTcF interval of >450 ms at screening, current treatment with medications that alter cardiac conduction (e.g., digitalis, β-blockers, or calcium channel blockers), ongoing adverse events (AEs) [National Cancer Institute Common Terminology Criteria for Adverse Events (NCI-CTCAE) grade ≥1] related to prior systemic therapy, surgery, or radiotherapy, peripheral sensory neuropathy with functional impairment (NCI-CTCAE grade >2) due to factors other than prior anticancer treatment, a life expectancy <12 weeks, trastuzumab therapy within 100 days, or any investigational agent within 28 days prior to the start of study treatment.

### Study treatment

Docetaxel and carboplatin were both administered intravenously on Cycle 1, Day 1, at respective doses of 75 mg/m^2^ and target area under the concentration–time curve (AUC) of 6 mg/ml/min [18]. Docetaxel and carboplatin continued to be administered on Day 1 of each treatment cycle, every 3 weeks (q3w).

**Fig. 1:**
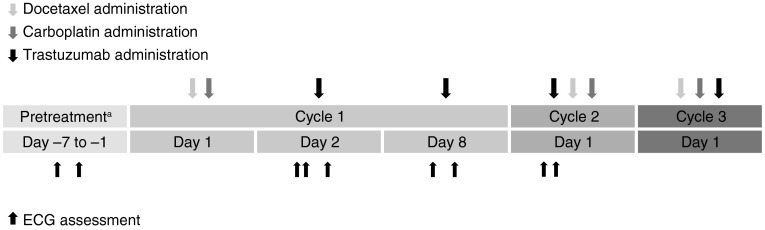
Schematic illustration for schedule of study drug administration and electrocardiogram assessments. *ECG* electrocardiogram. ^a^ECG assessments during the pretreatment period were made relative to the approximate time of future trastuzumab administration. On-study ECG assessments—Cycle 1, Day 2: 30 (±15) and 15 (±15) min preinfusion, 30 (±15) min postinfusion; Cycle 1, Day 8: 15 (±15) min preinfusion, 30 (±15) min postinfusion; Cycle 2, Day 1: 15 (±15) min preinfusion, 30 (±15) min postinfusion

Patients received an unapproved (off-label), accelerated trastuzumab loading dose regimen. Trastuzumab was administered intravenously at 6 mg/kg on Cycle 1, Day 2 and Cycle 1, Day 8, followed by a maintenance dose of 6 mg/kg on Day 1 of each subsequent q3w treatment cycle (Fig. 1). This novel regimen used an accelerated loading dose to achieve steady-state trastuzumab concentrations earlier (by Cycle 2) and to provide higher serum concentrations, to allow effective assessment of QT effects, in addition to the pharmacokinetic evaluation. The accelerated trastuzumab loading dose regimen had been previously studied in a phase I/II trial in which women with HER2-positive MBC were administered 6 mg/kg of trastuzumab on Days 1, 8, and 15 of the first q3w treatment cycle and then on Day 1 of each subsequent q3w cycle [19]. This regimen resulted in trough serum concentrations at the end of Cycle 1 (119 mg/l) which exceeded the steady-state trough concentrations (50.1 mg/l) achieved with the standard trastuzumab regimen (8 mg/kg loading dose, 6 mg/kg q3w maintenance dose). There were no new or unexpected toxicities, and the regimen was well tolerated [19].

All study treatment was given until disease progression (per investigator assessment), unacceptable toxicity, or for up to 12 months after the last patient had enrolled in the study, whichever came first. Patients were considered to have completed the study once they had received three cycles of study treatment or, for patients who continued beyond Cycle 3, once they had completed treatment (trastuzumab and/or chemotherapy), at the discretion of the investigator. No trastuzumab dose reductions were allowed. Dose delays of no more than two cycles were allowed for AEs; in the event of dose delays for more than two cycles, trastuzumab had to be discontinued. Dose delays and modifications for carboplatin and docetaxel were allowed as per their respective prescribing information. The study was conducted in accordance with the principles of the Declaration of Helsinki and Good Clinical Practice. The protocol was approved by the institutional review board/ethics committee of each site and all patients provided written, informed consent.

Because of the known risk of QT prolongation from 5-hydroxytryptamine type 3 (5-HT3) receptor antagonists, 5-HT3 antiemetics (e.g., granisetron, ondansetron) and other QT-prolonging drugs were prohibited on Cycle 1, Day 2, Cycle 1, Day 8, and Cycle 2, Day 1, between the trastuzumab preinfusion and postinfusion ECG assessments. Antiemetics or other drugs with a risk of QT prolongation and a long half-life (≥4 h) were also prohibited on days prior to Cycle 1, Day 2, Cycle 1, Day 8, and Cycle 2, Day 1. Because of its influence on QT interval variability, nicotine was not allowed in any form from Cycle 1, Day 1 through Cycle 2, Day 1, inclusive. Alternative antiemetic drugs without a known risk of QT prolongation (e.g., aprepitant with dexamethasone, or lorazepam) were permitted at the investigator’s discretion and per each drug’s prescribing information.

### ECG assessments

Triplicate 12-lead ECG readings were taken over a period of 2 min at each ECG assessment time point. The average of the triplicate ECG readings for each time point was used in the analysis. Two ECG assessments were performed during the pretreatment period (Study Day −7 to −1) (for validation of equipment), and then, ECG assessments were performed at the following on-study time points (Fig. 1): Cycle 1, Day 2, 30 (±15) min and 15 (±15) min pretrastuzumab infusion and 30 (±15) min postinfusion; Cycle 1, Day 8 and Cycle 2, Day 1, 15 (±15) min preinfusion and 30 (±15) min postinfusion.

Serum potassium, magnesium, and calcium levels had to be NCI-CTCAE grade ≤1 (as determined by local laboratory testing) before performing ECGs; if electrolyte levels were grade >1, patients were to receive electrolyte supplements, per standard institutional practice, to reduce levels to grade ≤1 prior to perform the ECG. Retesting of potassium, magnesium, and calcium levels was performed according to standard institutional practice.

A technically qualified central ECG laboratory (Cardiocore, Bethesda, MD, USA) was used to conduct blinded ECG readings and interpretation and to provide independent cardiology services, including consultation with investigators about patients with prolonged QTc intervals, supplying study sites with uniform, calibrated ECG machines capable of digital data transmission, and training technical site staff to ensure consistency in ECG acquisitions. All ECGs for a patient were performed on the same machine. The lead for interval readings was prespecified, and baseline and on-treatment ECG readings were based on the same lead. ECG readings were performed before collecting any corresponding pharmacokinetic samples. ECG readers were blinded to treatment details, time, and day identifiers. All ECG readings from a particular patient were reviewed by a single reader on 1 day.

### ECG analysis

The ECG analysis was performed in the ECG-evaluable patient population, which consisted of all patients who: (1) had received any trastuzumab doses; (2) had at least one interpretable baseline ECG assessment; (3) had at least one interpretable ECG assessment performed at steady-state trastuzumab concentration; and (4) had no infusion reaction requiring treatment.

The average of the two ECG assessments performed prior to the first trastuzumab infusion (on Cycle 1, Day 2) was used as the baseline ECG measurement. The off-label, accelerated trastuzumab loading dose regimen in this study was used to achieve earlier steady-state trastuzumab concentrations, such that ECG assessments on Cycle 1, Day 8 and Cycle 2, Day 1 were made at steady-state trastuzumab concentrations; thus, the average of Cycle 1, Day 8 and Cycle 2, Day 1 postinfusion ECG assessments was used as the ECG measurement at steady-state trastuzumab concentrations. Since QT intervals are inversely correlated with heart rate, QT was corrected using Fridericia’s correction (QTcF = QT/RR_0.33_). In addition, secondary analyses were performed using Bazett’s correction (QTcB = QT/RR_0.5_). Ninety percent two-sided confidence intervals (CIs) for QTcF and QTcB intervals were calculated, in line with International Conference on Harmonization (ICH) E14 guidance [13]; an upper bound of the 90 % two-sided CI of ≥20 ms was considered the threshold for clinical concern [20]. The proportions of patients at each time point within absolute QTc interval categories, as defined by ICH E14 guidance, were summarized. Secondary ECG parameters (heart rate, PR, RR, and QRS intervals) were also investigated.

All study treatment administration, ECG readings, and pharmacokinetic sampling were to be performed at approximately the same time of day and >1 h postprandial, to minimize variations due to circadian rhythms or eating, and to ensure that baseline and postdose ECGs were time window matched.

An exploratory analysis was carried out to assess any potential correlation between serum trastuzumab concentration and the change from baseline in QTcF interval. Individual baseline-adjusted QTcF values were plotted against serum trastuzumab concentrations from corresponding pharmacokinetic samples. A least-squares model (with no stratification for patient or cycle) was fit to the data, and the gradient of the line of linear fit was derived.

### Safety assessments

After initiation of study medications, all AEs and serious AEs, regardless of causality, were reported until the study completion/early termination visit (28–42 days after the last dose of study treatment). After this period, investigators only reported serious AEs that were considered to be related to study treatment. AEs were elicited and recorded at each study visit. All AEs were summarized by System Organ Class, preferred term, and NCI-CTCAE version 3.0 severity grade. LVEF was monitored by echocardiography and/or multiple-gated acquisition every 12 weeks, or more often if clinically indicated.

## Results

Between July 6, 2009 and April 11, 2012, 59 patients from 14 US sites enrolled in the study. At the clinical cutoff date of February 20, 2013, 43 patients (72.9 %) had received ≥3 cycles of study treatment, six patients (10.2 %) had discontinued after treatment at Cycle 3 but before completing the cycle, 37 patients (62.7 %) had discontinued after completing Cycle 3, and 16 patients (27.1 %) had discontinued before Cycle 3 (including one patient who received no study treatment). Since one patient received no study treatment, there were 58 safety-evaluable patients. One patient withdrew on Cycle 1, Day 2, prior to receiving trastuzumab, and four patients had missing postinfusion ECG data. Therefore, the ECG-evaluable population consisted of 53 patients.

An overview of patient demographics and baseline disease characteristics is provided in Supplementary Table 1. Patients had a median age of 61 and an Eastern Cooperative Oncology Group performance status of 1 (50.8 %) or 0 (49.2 %). Nine patients (15.3 %) had received prior anthracycline-based chemotherapy.

5-HT3 receptor antagonist antiemetics and a number of other drugs are known to prolong the QT/QTc interval. Despite these drugs being prohibited in this study, patients who received restricted concomitant medications (protocol violators) were not excluded from the ECG-evaluable population. Prior to ECG data collection, 38 of the 53 ECG-evaluable patients (71.7 %) received at least one restricted concomitant medication with a known risk of QT prolongation. The most frequently reported restricted concomitant medications were diphenhydramine [29 patients (54.7 %)], ondansetron hydrochloride [14 (26.4 %)], and prochlorperazine [12 (22.6 %)].

### QTc results

QT interval duration was negatively correlated with heart rate and positively correlated with RR interval duration, as shown in Supplementary Fig. S1. QT intervals were corrected using Fridericia’s (QTcF) and Bazett’s (QTcB) methods. Using Fridericia’s correction, the effects of heart rate and RR interval on QT interval were reduced, as shown by plotting individual average QTcF intervals against time-matched heart rates and RR intervals (Supplementary Fig. S1). This produced linear-fitted lines with small gradients, thus demonstrating no apparent relationship between QTcF interval and heart rate or RR interval, and thereby confirming the suitability of the QTcF correction method. In contrast, more variability was observed when using Bazett’s correction, with QTcB intervals overcorrected at higher heart rates and undercorrected at lower heart rates (Supplementary Fig. S1).

**Fig. 2:**
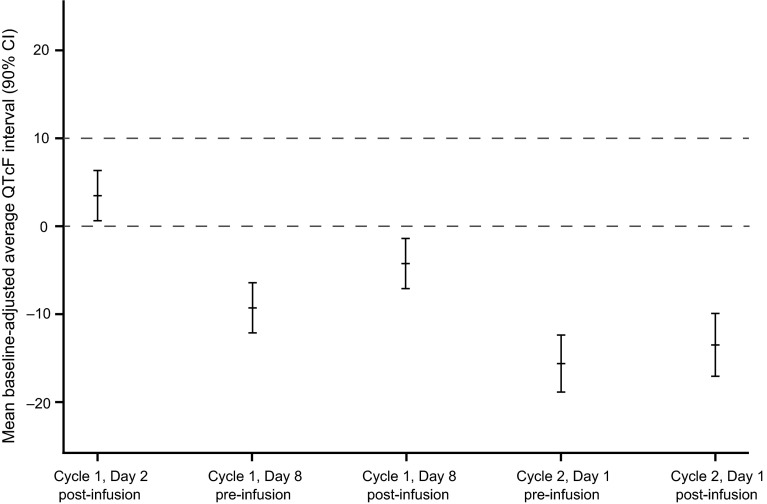
Mean baseline-adjusted QTcF interval (90 % two-sided confidence interval) by nominal electrocardiogram time point. *CI* confidence interval, *QTcF* QT interval corrected using Fridericia’s correction

The mean baseline-adjusted (i.e., the change from baseline) average QTcF interval (90 % two-sided CI) at each ECG assessment time point is shown in Fig. 2. For all time points, the upper bound of the 90 % two-sided CI was <10 ms. At 30 min postinfusion on Cycle 1, Day 2, the mean baseline-adjusted average QTcF interval was increased by 3.5 ms. At all other time points, the mean baseline-adjusted average QTcF intervals were reduced, with the lowest values occurring at Cycle 2, Day 1 pre- and postinfusion time points (−15.6 and −13.4 ms, respectively). Box plots of baseline-adjusted average QTcF intervals are shown in Supplementary Fig. S2. The majority of ECG readings were collected within the protocol-defined time window.

At steady-state trastuzumab concentrations (the average of postinfusion ECG assessments on Cycle 1, Day 8 and Cycle 2, Day 1), the mean baseline-adjusted QTcF interval was −8.4 ms (90 % CI −11.1, −5.7). Similarly, the mean baseline-adjusted QTcB interval at trastuzumab steady-state was −5.9 ms (90 % CI −9.2, −2.6).

The proportions of patients within absolute QTc interval categories, as defined by ICH E14 guidance, at each time point are presented in Table 1. No patient exhibited an absolute QTcF interval of >480 ms. Five patients at Cycle 1, Day 2 postinfusion, one patient at Cycle 1, Day 8 preinfusion, and two patients at Cycle 1, Day 8 postinfusion had absolute QTcF intervals between >450 and ≤480 ms. Compared with the QTcF interval, more patients had an absolute QTcB interval of >450 ms. At Cycle 1, Day 2 postinfusion, one patient had an absolute QTcB interval of >500 ms and two patients had absolute QTcB intervals between >480 and ≤500 ms. At Cycle 1, Day 8, two patients had absolute QTcB intervals between >480 and ≤500 ms (one preinfusion and one postinfusion). No patient had a baseline-adjusted QTcF interval of >30 ms, while one patient had a baseline-adjusted QTcB interval of >30 to ≤60 ms (at Cycle 1, Day 8, 30 min postinfusion). These results reflect the different correction factor used with Bazett’s method, which is generally acknowledged as a less ideal correction compared with Fridericia’s method.

**Table 1:** QTc intervals by nominal electrocardiogram time point based on ICH E14 guidance

ECG measure	ECG time point	*n* ^a^	Patients, *n* (%)			
			≤450 ms	>450–≤480 ms	>480–≤500 ms	>500 ms
QTcF	Cycle 1, Day 2					
	30 min postinfusion	49	44 (89.8)	5 (10.2)	0	0
	Cycle 1, Day 8					
	15 min preinfusion	50	49 (98.0)	1 (2.0)	0	0
	30 min postinfusion	50	48 (96.0)	2 (4.0)	0	0
	Cycle 2, Day 1					
	15 min preinfusion	48	48 (100)	0	0	0
	30 min postinfusion	48	48 (100)	0	0	0
QTcB	Cycle 1, Day 2					
	30 min post-infusion	49	36 (73.5)	10 (20.4)	2 (4.1)	1 (2.0)
	Cycle 1, Day 8					
	15 min preinfusion	50	41 (82.0)	8 (16.0)	1 (2.0)	0
	30 min postinfusion	50	39 (78.0)	10 (20.0)	1 (2.0)	0
	Cycle 2, Day 1					
	15 min preinfusion	48	43 (89.6)	5 (10.4)	0	0
	30 min postinfusion	48	43 (89.6)	5 (10.4)	0	0

*ECG* electrocardiogram, *ICH* international conference on harmonization, *QTcB* QT interval corrected using Bazett’s correction, *QTcF* QT interval corrected using Fridericia’s correction

^a^Number of patients with data available at that time point

The mean baseline-adjusted average PR interval increased over time to a maximum of 13.7 ms at the Cycle 2, Day 1, 30 min postinfusion time point. Overall, however, baseline-adjusted secondary ECG parameters showed no obvious trends that would suggest a clinical risk of cardiac dysrhythmia (Table 2). One patient’s PR interval duration was increased from baseline by ≥25 % and to an absolute value >200 ms at three time points; however, this patient experienced no cardiac or other relevant AEs; no patient had a QRS interval duration increased from baseline by ≥25 %. No patient had significant or nonsignificant abnormal U-wave changes from baseline and no patient had significant abnormal T-wave changes from baseline; the maximum number of patients with nonsignificant abnormal T-wave changes from baseline at any time point during the study was six (at Cycle 1, Day 8, 30 min postinfusion).

**Table 2:** Summary statistics for baseline-adjusted secondary electrocardiogram measures

ECG time point	QTcF (ms)	QTcB (ms)	QT (ms)	PR (ms)	QRS (ms)	RR (ms)	Heart rate (bpm)
Cycle 1, Day 2 30 min postinfusion (*n* = 49)							
Mean (SD)	3.5 (11.8)	3.9 (9.7)	2.9 (24.5)	−0.1 (9.4)	−0.1 (4.0)	0.3 (102.9)	0.5 (9.9)
Median	3.8	3.7	2.3	0.5	−0.7	0.0	0.2
Range	−30.8 to 28.2	−18.5 to 23.2	−67.3 to 50.3	−25.7 to 37.8	−10.3 to 9.0	−246.0 to 219.8	−16.5 to 31.7
Cycle 1, Day 8 15 min preinfusion (*n* = 50)							
Mean (SD)	−9.3 (12.1)	−0.8 (14.5)	−24.3 (29.0)	1.4 (14.6)	−1.8 (5.5)	−99.7 (144.1)	9.6 (14.9)
Median	−6.3	1.0	−19.5	−1.5	−1.5	−94.0	8.1
Range	−47.3 to 10.3	−41.3 to 24.8	−103.0 to 26.3	−34.0 to 61.8	−18.8 to 10.8	−524.0 to 167.5	−18.2 to 40.8
Cycle 1, Day 8 30 min postinfusion (*n* = 50)							
Mean (SD)	−4.3 (12.0)	1.4 (14.6)	−14.7 (27.5)	4.2 (11.8)	−1.8 (5.8)	−69.1 (137.8)	6.2 (13.7)
Median	−4.6	1.3	−8.5	2.3	−1.3	−55.2	4.2
Range	−28.2 to 24.7	−28.0 to 31.7	−96.7 to 29.7	−15.3 to 45.5	−20.8 to 11.3	−522.0 to 198.2	−20.8 to 38.8
Cycle 2, Day 1 15 min preinfusion (*n* = 48)							
Mean (SD)	−15.6 (13.3)	−13.2 (16.6)	−20.3 (27.0)	9.7 (13.1)	−0.5 (5.0)	−39.9 (139.3)	3.1 (12.7)
Median	−15.9	−14.8	−17.7	7.4	0.3	−30.8	2.8
Range	−43.8 to 7.8	−53.5 to 21.0	−79.3 to 40.3	−14.8 to 70.8	−16.0 to 7.8	−304.0 to 271.7	−22.3 to 28.0
Cycle 2, Day 1 30 min postinfusion (*n* = 48)							
Mean (SD)	−13.4 (14.6)	−14.2 (18.6)	−12.6 (26.1)	13.7 (16.1)	−0.3 (6.0)	−3.4 (136.7)	−0.7 (12.1)
Median	−12.2	−12.3	−12.1	13.3	0.4	−13.3	0.8
Range	−45.0 to 11.2	−59.8 to 28.3	−76.3 to 38.7	−12.8 to 92.5	−22.0 to 10.3	−283.0 to 368.0	−30.8 to 23.7

*n* is the number of patients with data available at that time point

*bpm* beats per minute, *ECG* electrocardiogram, *QTcB* QT interval corrected using Bazett’s correction, *QTcF* QT interval corrected using Fridericia’s correction, *SD* standard deviation

**Fig. 3:**
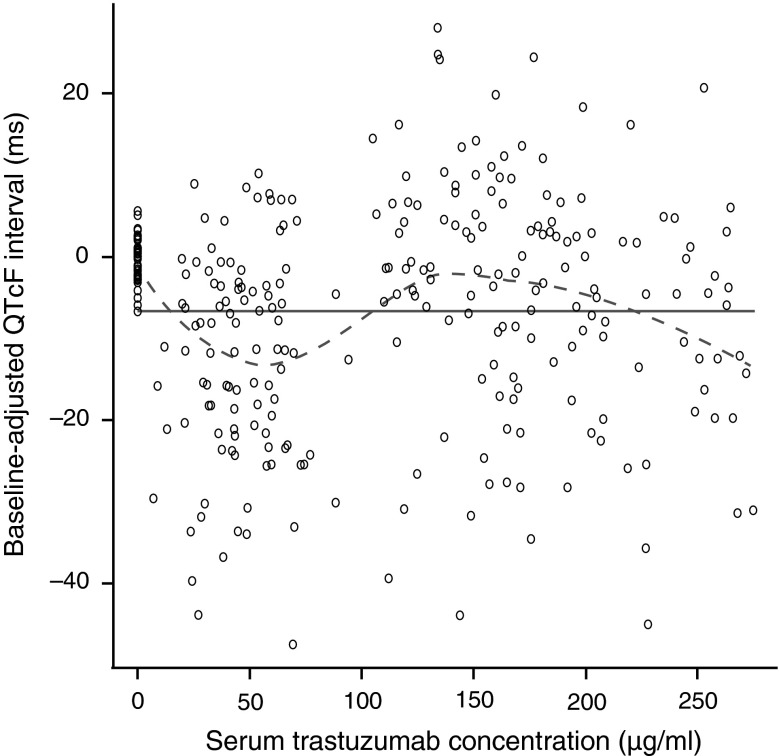
Relationship between baseline-adjusted QTcF interval and serum trastuzumab concentration. *QTcF* QT interval corrected using Fridericia’s correction. A least-squares model (with no stratification for patient or cycle) was fit to the data, with line of linear fit (*solid line*, slope = 0.0009, *p* = 0.926) and smoothed curve (*dotted line*, fitted using the S-Plus LOESS function)

An exploratory analysis was performed to assess whether any relationship existed between trastuzumab concentration and QTc interval. The Cycle 1, Day 2 preinfusion mean (95 % CI) trastuzumab concentration was 0.2 (0.2, 0.2) μg/ml; the mean Cycle 1, Day 8 and Cycle 2, Day 1 postinfusion concentrations were 186.4 (172.2, 201.7) and 179.1 (166.8, 192.4) μg/ml, respectively. The concentration–QTc dataset consisted of 297 ECG assessments with parallel trastuzumab concentration measurements from 53 patients, and contained data collected at trough and peak steady-state trastuzumab concentrations. A linear least-squares fit of trastuzumab concentration versus baseline-adjusted QTcF interval produced a slope of linear fit with a small gradient, close to 0 (0.0009), indicating no apparent relationship between trastuzumab concentration and baseline-adjusted QTcF interval (Fig. 3).

### Safety results

At data cutoff, the median duration of follow-up on study (the time from enrollment to the last on-study assessment) was 4.4 months (range 0.0–37.7 months). Patients had received a median of seven (range 1–52), four (range 1–24), and four (range 1–51) doses of trastuzumab, carboplatin, and docetaxel, respectively, and the median duration of trastuzumab treatment was 15 weeks (range 0–163 weeks). Fifty-seven out of 58 safety-evaluable patients had received at least one dose of trastuzumab.

A total of 57 out of 58 safety-evaluable patients (98.3 %) experienced AEs of any grade, and 49 patients (84.5 %) had experienced grade ≥3 AEs, the most common of which (in ≥4 patients) are shown in Table 3. Serious AEs were reported by 26 patients (44.8 %). Six patients (10.3 %) discontinued trastuzumab due to AEs, none of which were cardiac-related; two of these patients withdrew prematurely, before completing Cycle 3. Two patients (3.4 %) died during the study. One death was due to an AE of hemorrhagic stroke (considered by the investigator to be unrelated to trastuzumab), and the other was due to disease progression.

**Table 3:** Overview of adverse events

	Patients, *n* (%)
Any adverse event	57 (98.3)
Any serious adverse event	26 (44.8)
Any grade ≥3 adverse event	49 (84.5)
Grade ≥3 adverse events in ≥4 patients	
Neutrophil count decreased	33 (56.9)
White blood cell count decreased	13 (22.4)
Hemoglobin decreased	9 (15.5)
Platelet count decreased	9 (15.5)
Dehydration	5 (8.6)
Diarrhea	5 (8.6)
Fatigue	5 (8.6)
Febrile neutropenia	5 (8.6)
Hyperglycemia	4 (6.9)

Eight patients experienced cardiac AEs. The mean LVEF at baseline was 64.0 % [standard deviation (SD): 8.6 %], and the mean lowest LVEF value postbaseline was 59.5 % (SD: 6.6 %). Forty-four patients (93.6 %) had LVEF values of ≥50 % at all postbaseline time points; no postbaseline values of <45 % were observed during the study. Two patients experienced grade 2 left ventricular dysfunction. One of these events, considered by the investigator to be related to trastuzumab, resolved after 21 days and had no associated ECG changes. The other left ventricular dysfunction event, considered to be unrelated to trastuzumab, was ongoing at the end of the study. The patient had a history of left bundle branch block and had experienced an AE of prolonged QT interval several weeks prior to the left ventricular dysfunction event. This patient’s prolonged QT interval was an isolated grade 1 AE. Prior to this event, the patient experienced grade 2 hypocalcemia but did not receive the protocol-required calcium supplementation (and was therefore a protocol violator).

## Discussion

The results of this study suggest that trastuzumab had no clinically relevant effect on the duration of the QTcF or QTcB intervals in patients with HER2-positive metastatic or locally advanced, inoperable solid tumors. At the first postinfusion ECG assessment, the mean QTcF interval was increased slightly from baseline; at all subsequent time points, mean QTcF intervals were reduced from baseline. No patients had absolute QTcF interval durations that were within the range of clinical concern, i.e., there were no absolute values of >480 ms or increases from baseline of >30 ms. Additional ECG parameters (heart rate, QT, PR, RR, and QRS intervals) were not altered. Importantly, sustained QT interval prolongation did not occur even though a substantial proportion of patients received at least one restricted concomitant medication with a known risk of prolonging the QT interval. Furthermore, there was no relationship observed between serum trastuzumab concentration and change from baseline in QTcF interval duration. It should be noted that this study employed an unapproved (off-label), accelerated trastuzumab loading dose regimen to achieve steady-state serum trastuzumab concentrations early, in order to test more rigorously for potential effects on QT interval.

Increased rates of cardiac dysfunction were an unexpected observation during the clinical development of trastuzumab, influencing the design of subsequent trials, in which LVEF was monitored more frequently and stricter cardiac eligibility criteria were applied [7, 21, 22]. Trastuzumab-related cardiac dysfunction, which is dose-independent and reversible in most patients [22, 23], is believed to be related to cardiomyocyte HER2 expression. HER2 is thought to play a critical role in cardiac development, cardiomyocyte repair and survival, and in modulating myocardial stress responses in the adult heart [24–28]. Despite these insights, the potential arrhythmogenic effect of trastuzumab required further exploration.

Previous reports evaluating QT interval during trastuzumab-based therapy have been conflicting. An observational study reported long-term treatment with trastuzumab to be associated with significantly prolonged QT interval. However, this study compared patients with HER2-positive and HER2-negative EBC. Furthermore, the results were confounded by the use of anthracycline-based chemotherapy, which is known to affect cardiac function [15]. In another study, no acute effects on cardiac repolarization were seen following trastuzumab infusion [16]. Data from clinical studies of two other HER2-targeted humanized monoclonal antibodies, trastuzumab emtansine (Kadcyla^®^, F. Hoffmann-La Roche, Basel, Switzerland) and pertuzumab (PERJETA^®^, F. Hoffmann-La Roche, Basel, Switzerland), have also demonstrated no effect on QT interval [29, 30]. The lack of an observed effect of trastuzumab on QT interval is consistent with the molecular mechanism through which pharmaceutical agents are thought to affect cardiac repolarization [17]. Small molecules can bind to human ether-à-go-go-related gene (hERG) ion channels, disrupting ionic current. In contrast, monoclonal antibodies, such as trastuzumab, have a relatively large molecular size and high target specificity and are therefore not expected to access the drug-binding site of hERG channels.

Evaluation of the potential effect of trastuzumab on QT interval using a thorough QT study, as defined by ICH E14 guidance [13], was not feasible in the context of our study, as with other oncology studies [31]. Trastuzumab, in combination with carboplatin and docetaxel, could not be administered to healthy volunteers due to the potential for toxicity, and supratherapeutic doses of trastuzumab could not be administered to patients, although it should be noted that the dosing regimen used in our study is the highest dose that has ever previously been administered to patients. Furthermore, a crossover study involving a washout period, and placebo treatment was not deemed ethical in patients with cancer. Despite these considerations, our study was designed to rigorously investigate QT interval; it employed a central ECG laboratory, well-calibrated ECG machines, blinded, independent reviewers, and time window-matched ECG assessments.

In this study, trastuzumab in combination with carboplatin and docetaxel had an acceptable safety profile which was comparable to the known safety profile of the trastuzumab, carboplatin, and docetaxel regimen in patients with HER2-positive EBC [32]. No QT prolongation was observed in patients with HER2-positive metastatic or locally advanced solid tumors when trastuzumab was administered as an accelerated loading dose regimen, in combination with carboplatin and docetaxel. Based on the results of this study, it is unlikely that trastuzumab administered via the approved dosing regimen would prolong the duration of the QT interval in clinical practice. Further exploration may be required to confirm this.

## Electronic supplementary material

Below is the link to the electronic supplementary material.

Supplementary material 1 (DOCX 13 kb)

**Supplementary Fig. S1** Uncorrected QT, QTcF, and QTcB intervals by heart rate and by RR interval. QTcB, QT interval corrected using Bazett’s correction; QTcF, QT interval corrected using Fridericia’s correction. (A) Uncorrected QT interval plotted against heart rate with line of linear fit (slope = –1.76, *p* < 0.001); (B) Uncorrected QT interval plotted against RR interval with line of linear fit (slope = 0.17, *p* < 0.001); (C) QTcF interval plotted against heart rate with line of linear fit (slope = –0.17, *p* = 0.032); (D) QTcF interval plotted against RR interval with line of linear fit (slope = 0.01, *p* = 0.081); (E) QTcB interval plotted against heart rate with line of linear fit (slope = 0.72, *p* < 0.001); (F) QTcB interval plotted against RR interval with line of linear fit (slope = –0.07, *p* < 0.001). (EPS 3069 kb)

**Supplementary Fig. S2** Box plots of baseline-adjusted QTcF interval by nominal electrocardiogram time point. QTcF, QT interval corrected using Fridericia’s correction. The standard span is 1.5× (interquartile range). Whiskers are drawn to the nearest value not beyond a standard span from the quartiles. Points beyond this (outliers) are drawn individually (EPS 811 kb)
