# Supplementary material for: Trastuzumab, in combination with carboplatin and docetaxel, does not prolong the QT interval of patients with HER2-positive metastatic or locally advanced inoperable solid tumors: results from a phase Ib study
Source: Cancer Chemother Pharmacol. 2014 Oct 26;74(6):1251–60. doi: 10.1007/s00280-014-2603-9 (PMC4236615; doi:10.1007/s00280-014-2603-9)
Supplement: Supplementary file 1 — Supplementary material 1 (DOCX 13 kb) [file 280_2014_2603_MOESM1_ESM.docx]

**Supplementary Table 1** Patient demographics and baseline disease characteristics

|  | All patients  *N* = 59 |
| --- | --- |
| Age, years  Median (range) | 61 (30–82) |
| Sex, *n* (%)  Male  Female | 26 (44.1)  33 (55.9) |
| Ethnicity, *n* (%)  Not Hispanic or Latino  Hispanic or Latino  Not available | 48 (81.4)  10 (16.9)  1 (1.7) |
| Race, *n* (%)  American Indian or Alaska Native  Black or African American  White  Not available | 4 (6.8)  6 (10.2)  44 (74.6)  5 (8.5) |
| ECOG score, *n* (%)  0  1 | 29 (49.2)  30 (50.8) |
| Baseline LVEF  Median (range) | 64 (50–93) |
| Prior cancer treatment, *n* (%)  Surgery  Radiation therapy  Anthracycline chemotherapy  Non-anthracycline chemotherapy  Hormonal therapy  Biologic therapy  Other | 50 (84.7)  31 (52.5)  9 (15.3)  51 (86.4)  7 (11.9)  15 (25.4)  15 (25.4) |

ECOG, Eastern Cooperative Oncology Group; LVEF, left ventricular ejection fraction
